# A Hybrid EMD-Kurtosis Method for Estimating Fetal Heart Rate from Continuous Doppler Signals

**DOI:** 10.3389/fphys.2017.00641

**Published:** 2017-08-30

**Authors:** Haitham M. Al-Angari, Yoshitaka Kimura, Leontios J. Hadjileontiadis, Ahsan H. Khandoker

**Affiliations:** ^1^Department of Biomedical Engineering, Khalifa University of Science and Technology Abu Dhabi, United Arab Emirates; ^2^The Graduate School of Medicine, Tohoku University Sendai, Japan; ^3^Department of Electrical and Computer Engineering, Khalifa University of Science and Technology Abu Dhabi, United Arab Emirates; ^4^Department of Electrical and Computer Engineering, Aristotle University of Thessaloniki Thessaloniki, Greece

**Keywords:** fetal heart rate, fetal Doppler ultrasound, autocorrelation, kurtosis, empirical mode decomposition

## Abstract

Monitoring of fetal heart rate (FHR) is an important measure of fetal wellbeing during the months of pregnancy. Previous works on estimating FHR variability from Doppler ultrasound (DUS) signal mainly through autocorrelation analysis showed low accuracy when compared with heart rate variability (HRV) computed from fetal electrocardiography (fECG). In this work, we proposed a method based on empirical mode decomposition (EMD) and the kurtosis statistics to estimate FHR and its variability from DUS. Comparison between estimated beat-to-beat intervals using the proposed method and the autocorrelation function (AF) with respect to RR intervals computed from fECG as the ground truth was done on DUS signals from 44 pregnant mothers in the early (20 cases) and late (24 cases) gestational weeks. The new EMD-kurtosis method showed significant lower error in estimating the number of beats in the early group (EMD-kurtosis: 2.2% vs. AF: 8.5%, *p* < 0.01, root mean squared error) and the late group (EMD-kurtosis: 2.9% vs. AF: 6.2%). The EMD-kurtosis method was also found to be better in estimating mean beat-to-beat with an average difference of 1.6 ms from true mean RR compared to 19.3 ms by using the AF method. However, the EMD-kurtosis performed worse than AF in estimating SNDD and RMSSD. The proposed EMD-kurtosis method is more robust than AF in low signal-to-noise ratio cases and can be used in a hybrid system to estimate beat-to-beat intervals from DUS. Further analysis to reduce the estimated beat-to-beat variability from the EMD-kurtosis method is needed.

## Introduction

Fetal Heart Rate (FHR) monitoring (1997) by Doppler based cardiotocography (CTG) in the third trimester is a commonly established method to identify fetal compromises. All pregnancies are usually checked by FHR monitor to identify any abnormality in FHR pattern (2001). The decrease of the FHR indicates an abnormal situation of the pregnancy particularly during uterine contraction (Peters et al., 2001). It has been shown however that modern fetal monitors using the Doppler US technique (DUS) do not provide reliable evaluation of FHR variability (Fuchs, 2014). This is due to the changing fetal Doppler signal over time as a result of location changes of fetal heart and the high signal to noise ratio (SNR) which make it very difficult to determine the beat-to-beat intervals (Shakespeare et al., 2001). Also, abdominal fetal electrocardiography (fECG) has been reported to provides more reliable description of the instantaneous FHR variability than DUS-based approaches due to the improvement in instrumentation, electrode technology and signal processing approaches related to detecting fECG from abdominal maternal ECG (Jezewski et al., 2017). Another limitation with the DUS techniques (in the currently available clinical systems) is that the estimated FHR typically resampled at 4 Hz which intrinsically dilutes the data stream of the short-term FHR fluctuations (Durosier et al., 2014; Li et al., 2015).

However, the important question still remains to be answered if it is possible to obtain beat-to-beat FHR reliably from fetal Doppler signals as compared to the same obtained by R–R intervals of fECG signals?

Various processing strategies have been tested on Doppler signals to generate FHR. For examples, band-pass filtering (Tuck, 1982; Spencer et al., 1987; Boos and Schraag, 1992) and the use of auto-correlation function (AF) (Tuck, 1982). However, it has been shown that the estimated heart rate variability (HRV) computed by the AF is inaccurate (Takeuchi and Hogaki, 1978; Divon et al., 1985). This is due to the averaging nature of the AF method where a single periodicity value is determined from all (neighboring) heart beats enclosed in the AF window (Lauersen et al., 1976; Cesarelli et al., 2009). Also, the AF uses the envelope of the DUS signal to determine the instantaneous periodicity which correspond to the cardiac cycle but not the consecutive heart beats. Recently an AF method with an adaptive window size has shown improved accuracy in estimating the beat-to-beat interval from DUS (Jezewski et al., 2011). Using that method, the cardiac cycles are represented by sets of periodicity measurements (computed from the autocorrelation of multiple shifts of a certain window size). Then, these periodicity measurements are segmented using a segmentation algorithm where the location of the successive segment is continuously adjusted and based on the measurements contained in each segment an estimated of the new cardiac cycle duration is computed.

The empirical mode decomposition (EMD) method decomposes a signal into components with well-defined instantaneous frequency. These components are called Intrinsic Mode Functions (IMFs). Each IMF has a unique local frequency and different IMFs do not exhibit the same frequency at the same time (Huang et al., 1998). The ensembled EEMD evolved from EMD to treats the problem of mode mixing where different modes of oscillation may appear in one IMF or one mode can spread across different IMFs (Wu and Huang, 2009). This make the EEMD a true filter for any data. Applying the kurtosis on the (filtered) IMFs is done to determine which parts of these IMFs is important (high kurtosis value) and unimportant (low kurtosis value). Kurtosis has been used previously in detecting waveform changes (Saragiotis et al., 2004; Rekanos and Hadjileontiadis, 2006). In the DUS case, the locations of the signal peaks are expected to be within these high kurtosis parts.

Therefore, in this study, we propose a method combining EMD and kurtosis to estimate FHR and fetal HRV from DUS signal. We compare FHR (mean, standard deviation, and root mean squared successive difference) values estimated from DUS using both the AF method and our proposed method with a true RR interval determined from fECG of 44 healthy fetuses from early and late gestational age groups.

## Materials and methods

### Subjects

Abdominal ECG and Doppler ultrasound signals (DUS) were collected from 51 pregnant women at Tohoku University Hospital in Japan. The pregnant women were lying on their backs while the abdominal ECG signals were collected using 12 electrodes: ten on the mother's abdomen, one reference electrode on the back and one electrode at the right thoracic position. Signals were recorded during daytime (between 10 a.m. and 4 p.m.) over 3 years (2009–2011). The same experimental set up was applied to all the pregnant mothers who participated in this study. The continuous DUS data were obtained using ultrasonic transducer 5,700 (fetal monitor 116, Corometrics Medical Systems, Inc.) with 1.15 MHz signals. The recordings were of 1-min length and were sampled at 1 kHz with 16-bit resolution. The continuous DUS was recorded in a laptop by a data acquisition system which synchronizes with Abdominal ECG machine. fECG signals were separated by another custom-made software. The study protocol was approved by Tohoku University Institutional Review Board (IRB: 2015-2-80-1) and written informed consent was obtained from all subjects. The inclusion criteria for the study were: (1) Signed on written consent form, (2) Maternal age of 20 years or older, and (3) gestational age in the range of 24–42 weeks. The exclusion criteria were: (1) Diagnosed with multiple pregnancy, abnormal pregnancy, pregnancy with an obstetric complication (e.g., gestational diabetes, gestational hypertension, uterine fibroids, and cervical cancer) and (2) Scheduled for Caesarean section. The raw ECG and DUS signals were visually checked and noisy records (visually no peaks were seen) were removed from the dataset (two records). The remaining records were divided into two age groups: early gestational group (≤32 weeks; 20 cases) and late gestational group (≥35 weeks; 24 cases). Records with gestational age in-between the two groups were removed from the analysis (five records).

### Empirical mode decomposition (EMD)

In this work, the EMD was used to analyzed the oscillatory behavior of DUS. The EMD method deals with non-stationary and non-linear data (Huang et al., 1998). The EMD method consider that a signal consists of different simple intrinsic modes of oscillations. These simple oscillations are represented by the IMFs which satisfy two conditions:

1. The number of zero-crossings and the number of extrema must either equal or differ at most by one in the whole dataset.
2. The mean value of the envelope defined by the local maxima and the envelope defined by the local minima is zero at any point.

A signal *x*(*t*) is represented by the EMD method as:

(1) $$\begin{array}{r} x\left(t\right)=\overset{N}{\underset{i=1}{\sum }}C_{i}\left(t\right)+r_{N}\left(t\right), \end{array}$$

where *c*_*i*_(t) is the *i*-th IMF and *r*_*N*_(t)the final residue.

### Estimation of R peak location

To separate fECG from the composite abdominal signal, a combination of maternal ECG cancelation and blind source separation with the reference signal (BSSR) was used (Sato et al., 2007). In brief, electrical activities of the heart can be modeled as a vector in the direction of excitation called the heart vector (Symonds et al., 2001). The maternal ECG component was excluded by subtracting the linear combination of mutually orthogonal projections of the heart vector. After that, BSSR which is a kind of neural network method, was used to extract fECG from complex mixture using DUS signal as a reference. RR interval (intervals between successive R waves of the fECG were then computed using the algorithm developed by Pan and Tompkins (1985). The computed RR intervals from the fECG (true RR) were considered, the ground truth to be compared with the estimated beat-to-beat intervals from the DUS using the combined EMD-kurtosis and the AF methods.

To estimate beat location from DUS using the combined EMD-kurtosis method, the background noise was first removed from the DUS signal using wavelet thresholding with the first 15 levels of Haar wavelet. The signal was then decomposed into its IMFs using the EEMD method.

The kurtosis defined as:

(2) $$\begin{array}{r} {\hat{\gamma }}_{4}=\left(N-1\right)\frac{\sum_{n=1}^{N}x^{4}\left(n\right)}{{\left(\sum_{n=1}^{N}x^{2}\left(n\right)\right)}^{2}} \end{array}$$

was computed for each IMF signal *x*(*n*) where *N* is the number of the sample in the signal. The kurtosis of each IMF was tested against the Chebyshev inequality to determine whether it was good or noisy for the detection of the DUS peak location. More details on the method can be found at (Papadaniil and Hadjileontiadis, 2014).

Then, for all selected IMF a sliding window with a width of 50–600 ms (with an increment of 50 ms) and a shift of 1 ms was used to compute the kurtosis at each shift point. A matrix of *I*x*W* kurtosis vectors was constructed where *I* indicate the number of selected IMF and *W* is the number of used windows. Again, each vector was tested against the Chebyshev inequality to be selected in the final subgroup used to estimate beat location. Two other measures were introduced to fine select the best kurtosis vectors:

1. The percentage of the mismatch error between true R peaks count (determined from fECG) and estimated beat count (determined by the peaks of the selected kurtosis vectors) as:
  (3) $$\begin{array}{r} mismatch\;error=\frac{true\;R\;count-estimated\;beat\;count}{true\;R\;count}\times 100 \end{array}$$
2. The variability of the estimated beat location (*var*B_loc) was determined by the standard deviation of the absolute difference between real R peak location and the estimated beat location:

A selected kurtosis vector would minimize both the mismatch error and *var*B_loc for better estimation of the beat location. Finally, the selected vectors were summed up and a peak search with minimum peak distance of 300 ms was performed.

The estimation of beat-to-beat intervals using AF method was done similar to (Jezewski et al., 2011). Two measures were used to compare results between the two methods. These were:

1. The absolute mismatch error.
2. The mean successive beat error, where the absolute beat-to-beat error was defined as:
  (4) $$\begin{array}{r} Successive\;beat\;error\;\left(i\right)=\\ \;\frac{\left|true\;RR\left(i\right)-estimated\;beat-to-beat\left(i\right)\right|}{true\;RR\left(i\right)}\;\times 100 \end{array}$$

for *i* = 1: *N*_*min*_ where *N*_*min*_ is minimum length of the two true RR and estimated beat-to-beat vectors.

Finally, a comparison between the true RR and estimated beat-to-beat (using AF and EMD-kurtosis methods) in the mean, standard deviation (SDNN) and root mean square of successive difference (RMSSD) was performed using Kruskal-Wallis test. Significant differences were reported if *p* < 0.05.

## Results

**Figure 1:**
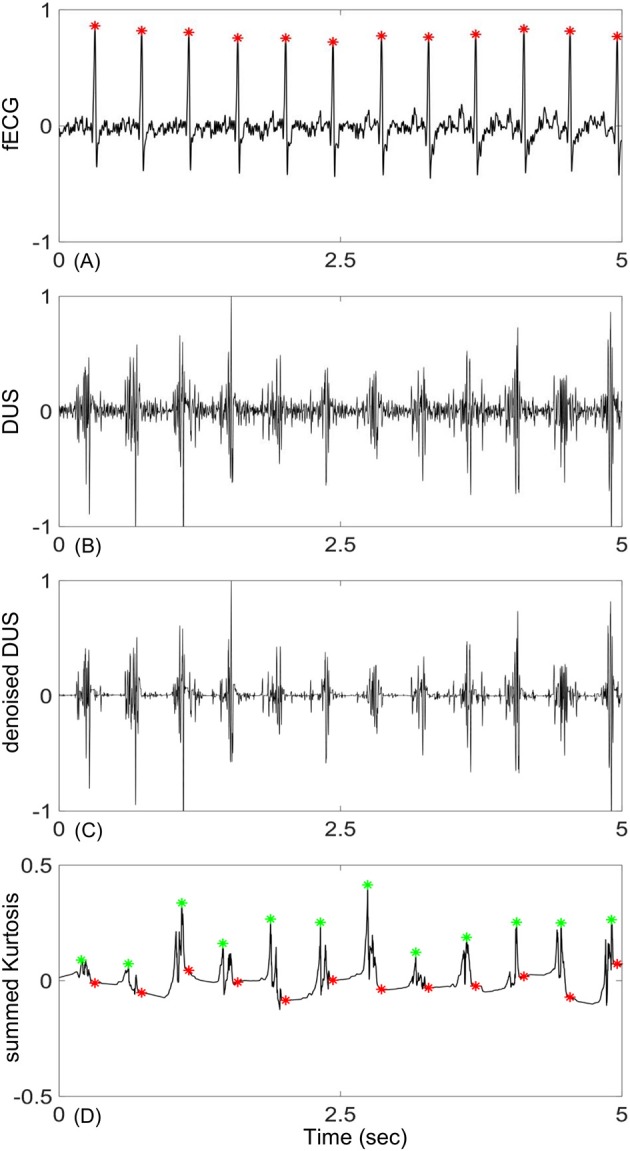
An example of fetal Doppler signal and the derived kurtosis signal: **(A)** 5-s segment of fetal ECG (fECG), **(B)** corresponding Doppler ultra sound signal (DUS), **(C)** DUS after noise removal using the first 15 levels of the Haar wavelet, **(D)** constructed kurtosis signal from the selected IMFs of DUS (green stars are location of the kurtosis peaks and red stars are true location of the R peaks determined from fECG). Signals were normalized to their maximum values.

**Figure 2:**
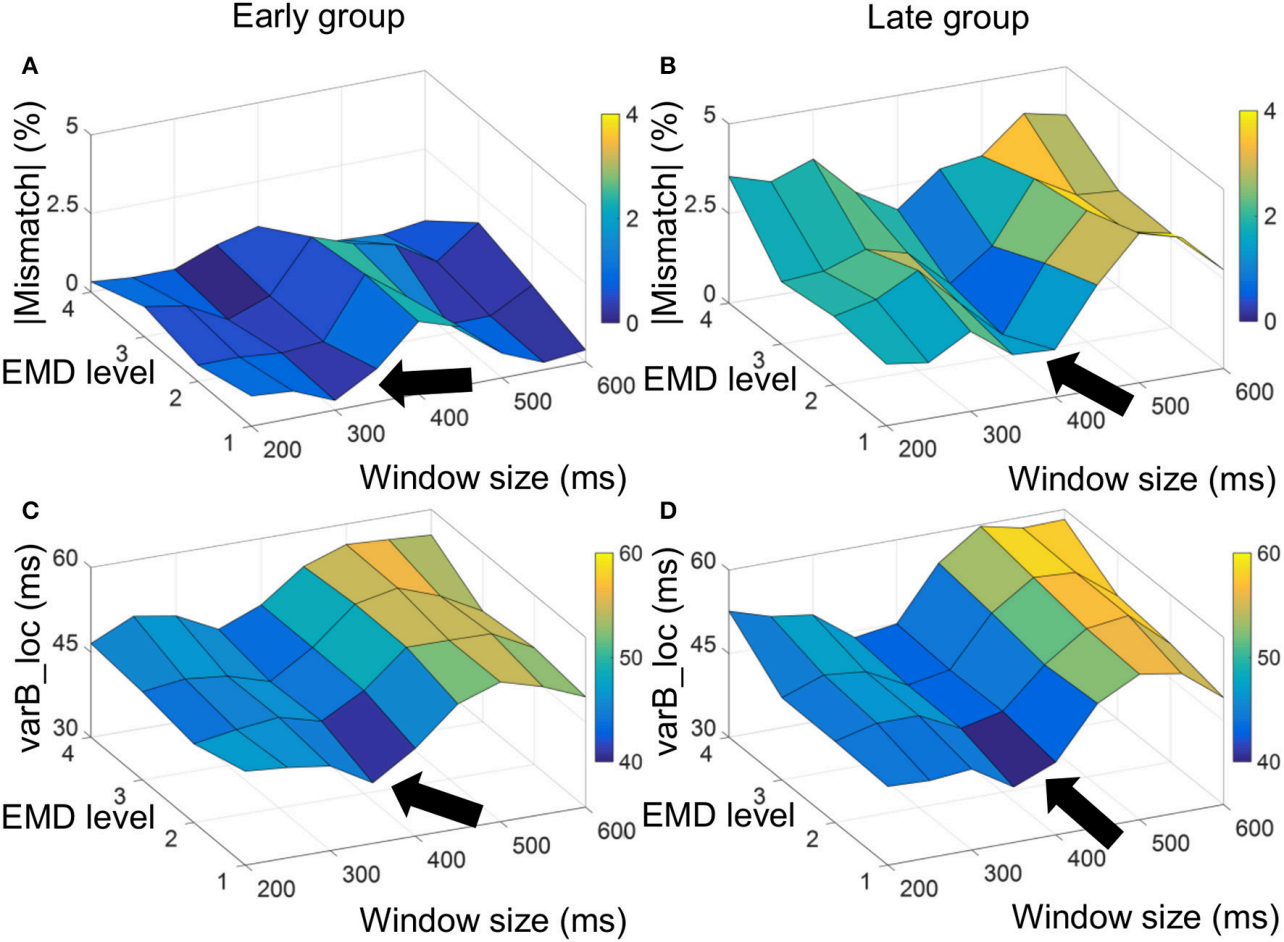
Absolute value of mismatch error and varB_loc measure used to optimize selection of window size to compute the EMD-kurtosis signal. For the early group the minimum absolute mismatch error and varB_loc were achieved at window size between 300 and 350 ms (**A,C**, respectively) and for the late group the minimum absolute mismatch error and varR_loc were achieved at window size between 350 and 400 ms (**B,D**, respectively). The arrow point at window sizes that minimize the measurements.

The mean ± Standard Deviation (SD) of the early group gestational age was 27 ± 4.3 weeks and for the late group was 38 ± 1.7 weeks. The RR interval duration of the early group was 406.4 ± 27 ms and for the late group was 422.3 ± 29 ms. An example of an extracted fECG with the corresponding DUS and the estimated beat location using the EMD-kurtosis method is shown in Figure 1. After noise removal from the raw DUS (Figures 1B,C), the IMFs were generated and the kurtosis of the IMFs with different window size was computed. The kurtosis signals with optimum window sizes and EMD levels were summed up (Figure 1D) and the peaks of the resultant signal were detected. The differences between the successive peak locations represented the estimated beat-to-beat intervals. The optimum EMD levels and window sizes were selected based on the criteria shown in Figure 2.

Figure 2 shows a surf plot of the average mismatch error and varB_loc for the early and late age groups. For the early group, the minimum mismatch error and varB_loc were achieved at window size around 300–350 ms and the first three EMD levels while for the late group the minimum was achieved around window size of 350–400 ms with same EMD levels. This smaller grid was used to compute the final (summed) kurtosis signal.

**Figure 3:**
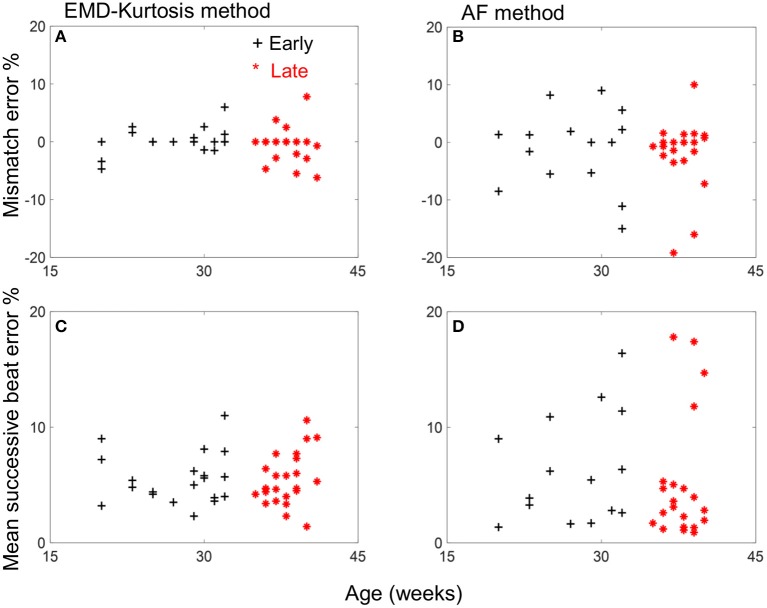
Mismatch and mean successive beat error vs. age for the early group (black +) and late group (red *) cases for the EMD-kurtosis method **(A,C)** and the AF method **(B,D)**. Negative sign of mismatch error indicates more estimated beats than the true value.

Table 1 shows a quantified comparison between the EMD-kurtosis and AF methods. Both the mismatch and mean successive beat errors were lower using EMD-kurtosis for both early and late groups. This can also be observed from Figure 3. The mismatch error was significantly lower using EMD-kurtosis for the early group (*p* < 0.01) and the mean successive beat error was significantly lower using EMD-kurtosis in the late group (*p* < 0.05). No significant was found when comparing early and late groups using the same method. Figure 3 shows the % mismatch and mean absolute beat-to-beat error for all cases in the early and late groups. Generally speaking, these measures were lower than the 10% error limit for the EMD-kurtosis method while for the AF method some case exceeded that limit. % mismatch error was clearly higher using AF in the early group (Figures 3A,B). Four cases in the late group were having higher absolute beat-to-beat error compared to the other cases in the group using AF which was not observed using the EMD-kurtosis method (Figures 3C,D).

**Table 1:** Comparison between the EMD-kurtosis and the AF methods in mismatch error and mean successive beat error.

**Group**	**Number of cases**	**Age (weeks)**	**Mismatch error (%)**^‡^		**Mean successive beat error (%)**	
			**EMD-kurtosis**	**AF**	**EMD-kurtosis**	**AF**
Early	20	27 ± 4.3	2.2	8.5^†^	5.5 ± 2.2	7.3 ± 5.8
Late	24	38 ± 1.7	2.9	6.2	5.4 ± 2.2	5.3 ± 5.4^*^

*, † *AF is significantly higher than EMD-kurtosis with p < 0.05, p < 0.01, respectively*.

‡ *values represented in mean root squared*.

Table 2 shows a comparison of the mean, SDNN and RMSSD between the true RR and estimated beat-to-beat. The EMD-kurtosis method was better in estimating mean beat-to-beat while the estimated SNDD and RMSSD were significantly higher than the true RR values using both methods (the EMD-kurtosis showed higher values).

**Table 2:** Comparison between true RR and estimate beat-to-beat intervals using the EMD-kurtosis and the AF methods.

	**Mean beat-to-beat (ms)**		**SDNN (ms)**		**RMSSD (ms)**	
	**Early**	**Late**	**Early**	**Late**	**Early**	**Late**
True RR	406.4 ± 27	420 ± 27	13.5 ± 7.1	16.5 ± 13.4	9.5 ± 11.7	9.7 ± 11.1
Estimated beat-to-beat EMD-kurtosis	406.3 ± 23	417 ± 21	30.1 ± 8.8^†^	31.8 ± 10.3^†^	27.5 ± 6.3^†^	29.1 ± 6.5^†^
Estimated beat-to-beat AF	386.6 ± 27	402 ± 34	23.9 ± 10.6^†^	23.5 ± 9.3^†^	18.8 ± 5.5^†^	16.6 ± 4.8^†^

† *Significantly different from true RR (p < 0.01). Kruskal-Wallis test was done between true RR parameters and estimated parameters from each of the EMD-kurtosis and the AF methods separately*.

**Figure 4:**
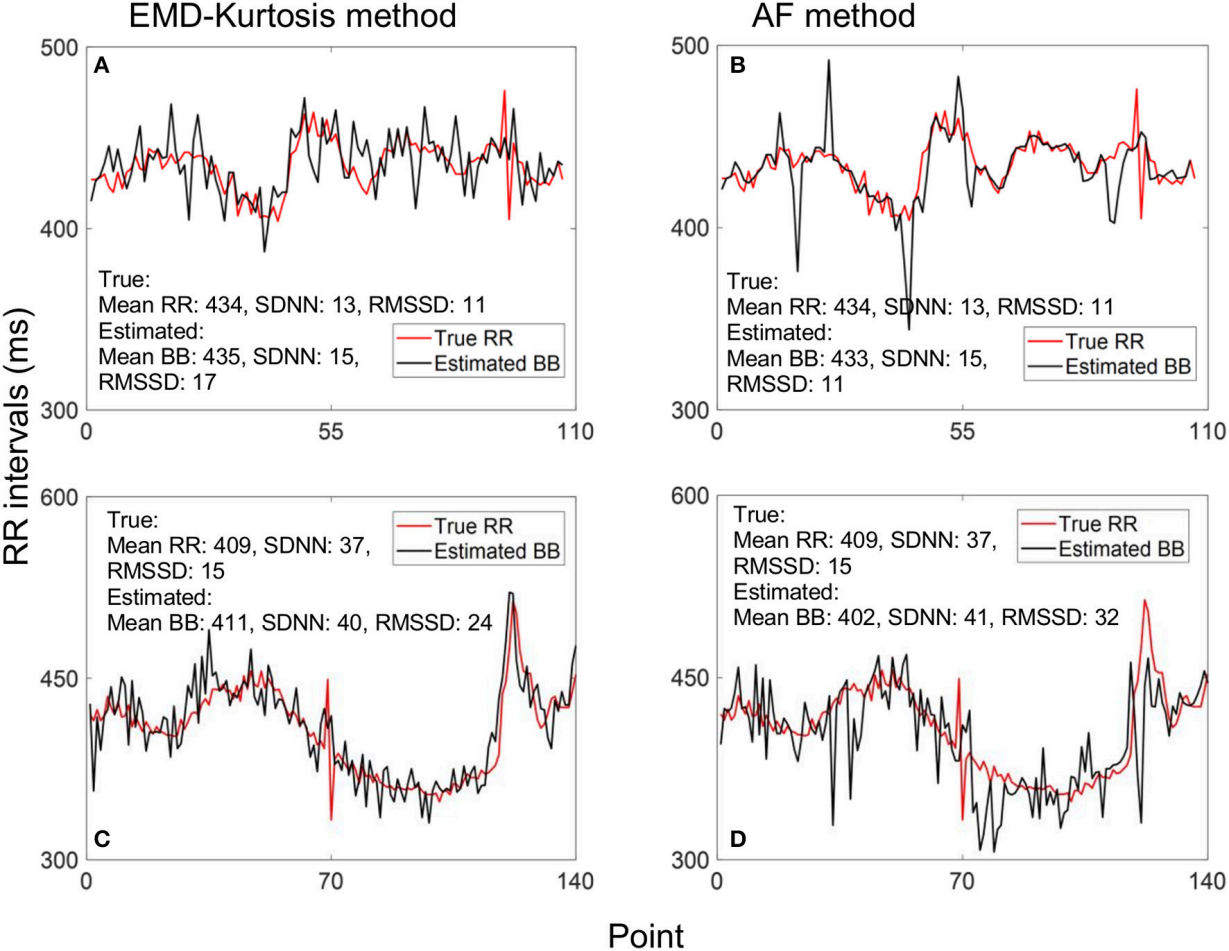
Example of estimating beat-to-beat (BB) intervals: **(A,C)** using the EMD-kurtosis method for early and late gestational weeks respectively, **(B,D)** using the autocorrelation function (AF) for the same cases in **(A,C)**. Red line indicate true RR interval computed from the fECG signal. For signals with lower variability the AF method showed better estimation **(A,B)** while the EMD-kurtosis method performed better for signals with higher RR variability **(C,D)**.

Figure 4 helps interpreting these results with examples of true RR and estimated beat-to-beat intervals for an early and a late case. When true RR variability is small the AF was better in estimating beat-to-beat intervals (Figures 4A,B) while the EMD-kurtosis method worked better with high RR variability.

**Figure 5:**
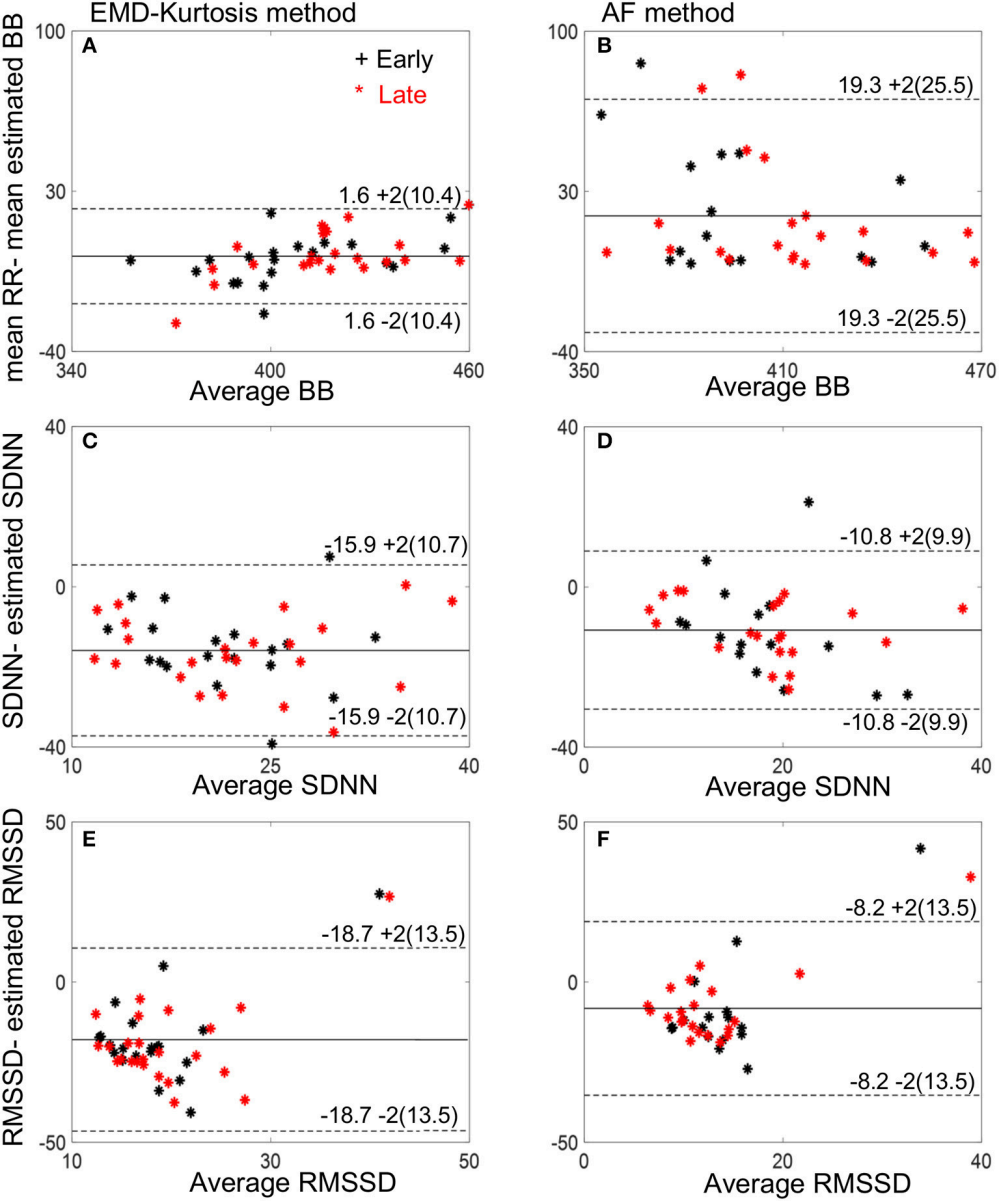
Bland-Altman plots of the estimated mean beat-to-beat (BB), SDNN and RMSSD using the EMD-kurtosis method **(A,C,E)** and the AF **(B,D,F)**. Black stars indicate early group cases and red stars indicate late group cases. Numbers next to the dashed lines represent mean ± 2SD of the true-estimated measures.

Bland-Altman plots of the estimated mean beat-to-beat, SDNN and RMSSD using the two methods compared to the true RR interval computed from fECG are shown in Figure 5. The EMD-kurtosis method has smaller mean difference between the true RR and the estimated beat-to-beat intervals mean where most of the cases were within the mean ± 2 SD range while the AF has smaller mean difference between true and estimated SDNN and RMSSD.

## Discussion

In this work, we have applied a method based on computing the kurtosis function on the IMFs extracted from the DUS signal to estimate cardiac beat-to-beat intervals. The proper choice of the window size used to compute the kurtosis was important in reducing the error in estimating both the number beats and the beat-to-beat variability. A window size slightly smaller than the duration of the mean RR interval provided the optimum results. This is obvious since a window of this size results in almost one distinguished kurtosis peak in a single cardiac cycle. Smaller or bigger windows increase the changes of missing true peaks in the situation of low SNR (the early group) and of having multiple peaks due to distinct walls and valves movements (the late group).

Due to developing fetal heart wall and valves in the early gestational week, the reflected Doppler signal is relatively weaker which reduces the SNR. This could be the reason of having higher error for AF method in the early gestational group (even when compared with the results of the same method for the late group). On the other hand, the EMD-kurtosis method was more robust against low SNR. It reduced the mismatch error four times for the early group and two times for the late group as compared to the AF method.

The mean successive beat error for the EMD-kurtosis method although was lower than AF but was still considered high (above 5%, Table 1) for good evaluation of HRV. Various reasons could explain this high error. Firstly, the DUS represents the mechanical movement of heart walls and valves while the R wave represents the electrical depolarization of the ventricle which results in an expected delay between the two signals. This delay could vary from beat to beat.

Also, heart valves movements which considered of high frequency cannot be completely separated from wall movements which have lower frequency which results in an increased variability in estimating HRV from the DUS signal. Other factors including movement of mother or baby and changing the orientation of the Doppler probe to fetal heart could also cause variability in estimating HRV.

Our proposed EMD-kurtosis method showed also improvement in estimating mean heart rate compared to AF in both early and late groups (Table 2). The beat-to-beat variability assessed by SDNN and RMSSD was significantly higher than the true RR intervals for both methods although it was higher for the EMD-kurtosis method. This could be due to the adaptive AF window that changes its size slowly which on one hand helps in reducing RR variability (Figure 4B) but on the other hand responds slowly to rapid changes in the true signal (Figure 4D) which results in low estimated mean.

A limitation of our study is that the collected data was of 1-min length which is too short to control the fetal states. However, short term recording is typically used for clinical investigation in pregnancy clinic. Changing the segment length from 5 to 2 min has shown changes in the RMSSD values for FHR estimated by fetal magnetocardiogram (fMCG) which is known to have higher temporal resolution than the DUS signal (Moraes et al., 2012). Longer signal durations (10–30 min) will be needed to verify these findings. The EMD-kurtosis algorithm is a time-domain method that can easily be implemented in a microprocessor in the same DUS machine or in a separate device for practical clinical use. One consideration will be the time needed to extract the IMFs especially with long signal duration. This work is considered a step toward good estimation of FHR and its variability from DUS signal. Further research combining the EMD-kurtosis with other non-linear signal processing methods are needed to reduce variability in beat-to-beat intervals estimation and improve the overall accuracy.

## Author contributions

HA, YK, LH, and AK designed the study approach. YK and AK designed the experiments. YK supervised data collection. HA was responsible for analysis and running statistics. HA, LH, and AK interpreted the results and wrote the manuscript.

### Conflict of interest statement

The authors declare that the research was conducted in the absence of any commercial or financial relationships that could be construed as a potential conflict of interest.

## References

[B1] (1997). Electronic fetal heart rate monitoring: research guidelines for interpretation. National institute of child health and human development research planning workshop. Am. J. Obstet. Gynecol. 177, 1385–1390. 9423739

[B2] (2001). Confidential Enquiry into Stillbirths and Deaths in Infancy(CESDI). Executive Summary of the 8th Annual Report, Maternal and Child Health Research Consortium, London.

[B3] Boos A. Schraag M. (1992). Method and Apparatus for Calculating the Fetal Heart Rate. U.S. Patent No 5,170,791 A Washington, DC: U.S. Patent and Trademark Office.

[B4] Cesarelli M. Romano M. Bifulco P. (2009). Comparison of short term variability indexes in cardiotocographic foetal monitoring. Comput. Biol. Med. 39, 106–118. 10.1016/j.compbiomed.2008.11.010 19193367

[B5] Divon M. Y. Torres F. P. Yeh S. Y. Paul R. H. (1985). Autocorrelation techniques in fetal monitoring. Am. J. Obstet. Gynecol. 151, 2–6. 10.1016/0002-9378(85)90413-2 3966502

[B6] Durosier L. D. Green G. Batkin I. Seely A. J. Ross M. G. Richardson B. S.. (2014). Sampling rate of heart rate variability impacts the ability to detect acidemia in ovine fetuses near-term. Front. Pediatr. 2:38. 10.3389/fped.2014.00038 24829897 PMC4017161

[B7] Fuchs T. (2014). Values of T/QRS ratios measured during normal and post-term pregnancies. J. Perinat. Med. 42, 349–357. 10.1515/jpm-2013-0181 24361770

[B8] Huang N. E. Shen Z. Long S. R. Wu M. C. Shih H. H. Zheng Q. (1998). The empirical mode decomposition and the Hilbert spectrum for nonlinear and non-stationary time series analysis. Proc. R. Soc. Lond. A Math. Phys. Eng. Sci. 454, 903–995. 10.1098/rspa.1998.0193

[B9] Jezewski J. Roj D. Wrobel J. Horoba K. (2011). A novel technique for fetal heart rate estimation from Doppler ultrasound signal. Biomed. Eng. 10:92. 10.1186/1475-925X-10-92 21999764 PMC3305903

[B10] Jezewski J. Wrobel J. Matonia A. Horoba K. Martinek R. Kupka T.. (2017). Is abdominal fetal electrocardiography an alternative to Doppler ultrasound for fhr variability evaluation? Front. Physiol. 8:305. 10.3389/fphys.2017.00305 28559852 PMC5432618

[B11] Lauersen N. H. Hochberg H. M. George M. E. (1976). Evaluation of the accuracy of a new ultrasonic fetal heart rate monitor. Am. J. Obstet. Gynecol. 125, 1125–1135. 10.1016/0002-9378(76)90819-X 952309

[B12] Li X. Xu Y. Herry C. Durosier L. D. Casati D. Stampalija T.. (2015). Sampling frequency of fetal heart rate impacts the ability to predict pH and BE at birth: a retrospective multi-cohort study. Physiol. Meas. 36, L1–L12. 10.1088/0967-3334/36/5/L1 25893461

[B13] Moraes E. R. Murta L. O. Baffa O. Wakai R. T. Comani S. (2012). Linear and nonlinear measures of fetal heart rate patterns evaluated on very short fetal magnetocardiograms. Physiol. Meas. 33, 1563–1583. 10.1088/0967-3334/33/10/1563 22945491 PMC3476048

[B14] Pan J. Tompkins W. J. (1985). A real-time QRS detection algorithm. IEEE Trans. Biomed. Eng. 32, 230–236. 10.1109/TBME.1985.325532 3997178

[B15] Papadaniil C. D. Hadjileontiadis L. J. (2014). Efficient heart sound segmentation and extraction using ensemble empirical mode decomposition and kurtosis features. IEEE J. Biomed. Health Inform. 18, 1138–1152. 10.1109/JBHI.2013.2294399 25014929

[B16] Peters M. Crowe J. Pieri J. F. Quartero H. Hayes-Gill B. James D.. (2001). Monitoring the fetal heart non-invasively: a review of methods. J. Perinat. Med. 29, 408–416. 10.1515/JPM.2001.057 11723842

[B17] Rekanos I. T. Hadjileontiadis L. J. (2006). An iterative kurtosis-based technique for the detection of nonstationary bioacoustic signals. Signal Process. 86, 3787–3795. 10.1016/j.sigpro.2006.03.020

[B18] Saragiotis C. D. Hadjileontiadis L. J. Rekanos I. T. Panas S. M. (2004). Automatic P phase picking using maximum kurtosis and kappa-statistics criteria. IEEE Geosci. Remote Sens. Lett. 1, 147–151. 10.1109/LGRS.2004.828915

[B19] Sato M. Kimura Y. Chida S. Ito T. Katayama N. Okamura K.. (2007). A novel extraction method of fetal electrocardiogram from the composite abdominal signal. IEEE Trans. Biomed. Eng. 54, 49–58. 10.1109/TBME.2006.883791 17260855

[B20] Shakespeare S. A. Crowe J. A. Hayes-Gill B. R. Bhogal K. James D. K. (2001). The information content of Doppler ultrasound signals from the fetal heart. Med. Biol. Eng. Comput. 39, 619–626. 10.1007/BF02345432 11804166

[B21] Spencer J. A. D. Belcher R. Dawes G. S. (1987). The influence of signal loss on the comparison between computer analyses of the fetal heart rate in labour using pulsed Doppler ultrasound (with autocorrelation) and simultaneous scalp electrocardiogram. Eur. J. Obstet. Gynecol. 25, 29–34. 10.1016/0028-2243(87)90089-X 3297840

[B22] Symonds E. Sahota D. Chang A. (2001). Fetal Electrocardiology. London: Imperial College Press.

[B23] Takeuchi Y. Hogaki M. (1978). An adaptive correlation ratemeter: a new method for Doppler fetal heart rate measurements. Ultrasonics 16, 127–137. 10.1016/0041-624X(78)90039-2 644684

[B24] Tuck D. L. (1982). Improvement in Doppler ultrasound human foetal heart rate records by signal correlation. Med. Biol. Eng. Comput. 20, 357–600. 10.1007/BF02442804 7109732

[B25] Wu Z. Huang N. E. (2009). Ensemble empirical mode decomposition: a noise-assisted data analysis method. Adv. Adap. Data Anal. 1, 1–41. 10.1142/S1793536909000047

